# Eukaryotic initiation factor 4B is a multi-functional RNA binding protein that regulates histone mRNAs

**DOI:** 10.1093/nar/gkae767

**Published:** 2024-09-03

**Authors:** Ana Quintas, Robert F Harvey, Emilie Horvilleur, Gavin D Garland, Tobias Schmidt, Lajos Kalmar, Veronica Dezi, Alberto Marini, Alexander M Fulton, Tuija A A Pöyry, Cameron H Cole, Martin Turner, Ritwick Sawarkar, Michael A Chapman, Martin Bushell, Anne E Willis

**Affiliations:** MRC Toxicology Unit, University of Cambridge, Gleeson Building, Tennis Court Road, Cambridge CB2 1QW, UK; MRC Toxicology Unit, University of Cambridge, Gleeson Building, Tennis Court Road, Cambridge CB2 1QW, UK; MRC Toxicology Unit, University of Cambridge, Gleeson Building, Tennis Court Road, Cambridge CB2 1QW, UK; MRC Toxicology Unit, University of Cambridge, Gleeson Building, Tennis Court Road, Cambridge CB2 1QW, UK; Cancer Research UK Beatson Institute, Garscube Estate, Switchback Road, Glasgow G61 1BD, UK; MRC Toxicology Unit, University of Cambridge, Gleeson Building, Tennis Court Road, Cambridge CB2 1QW, UK; MRC Toxicology Unit, University of Cambridge, Gleeson Building, Tennis Court Road, Cambridge CB2 1QW, UK; MRC Toxicology Unit, University of Cambridge, Gleeson Building, Tennis Court Road, Cambridge CB2 1QW, UK; MRC Toxicology Unit, University of Cambridge, Gleeson Building, Tennis Court Road, Cambridge CB2 1QW, UK; MRC Toxicology Unit, University of Cambridge, Gleeson Building, Tennis Court Road, Cambridge CB2 1QW, UK; MRC Toxicology Unit, University of Cambridge, Gleeson Building, Tennis Court Road, Cambridge CB2 1QW, UK; Immunology Programme, Babraham Institute, Babraham Science Campus, Cambridgeshire CB22 3AT, UK; MRC Toxicology Unit, University of Cambridge, Gleeson Building, Tennis Court Road, Cambridge CB2 1QW, UK; MRC Toxicology Unit, University of Cambridge, Gleeson Building, Tennis Court Road, Cambridge CB2 1QW, UK; Cancer Research UK Beatson Institute, Garscube Estate, Switchback Road, Glasgow G61 1BD, UK; MRC Toxicology Unit, University of Cambridge, Gleeson Building, Tennis Court Road, Cambridge CB2 1QW, UK

## Abstract

RNA binding proteins drive proliferation and tumorigenesis by regulating the translation and stability of specific subsets of messenger RNAs (mRNAs). We have investigated the role of eukaryotic initiation factor 4B (eIF4B) in this process and identify 10-fold more RNA binding sites for eIF4B in tumour cells from patients with diffuse large B-cell lymphoma compared to control B cells and, using individual-nucleotide resolution UV cross-linking and immunoprecipitation, find that eIF4B binds the entire length of mRNA transcripts. eIF4B stimulates the helicase activity of eIF4A, thereby promoting the unwinding of RNA structure within the 5′ untranslated regions of mRNAs. We have found that, in addition to its well-documented role in mRNA translation, eIF4B additionally interacts with proteins associated with RNA turnover, including UPF1 (up-frameshift protein 1), which plays a key role in histone mRNA degradation at the end of S phase. Consistent with these data, we locate an eIF4B binding site upstream of the stem–loop structure in histone mRNAs and show that decreased eIF4B expression alters histone mRNA turnover and delays cell cycle progression through S phase. Collectively, these data provide insight into how eIF4B promotes tumorigenesis.

## Graphical Abstract

**Graphical Abstract:**
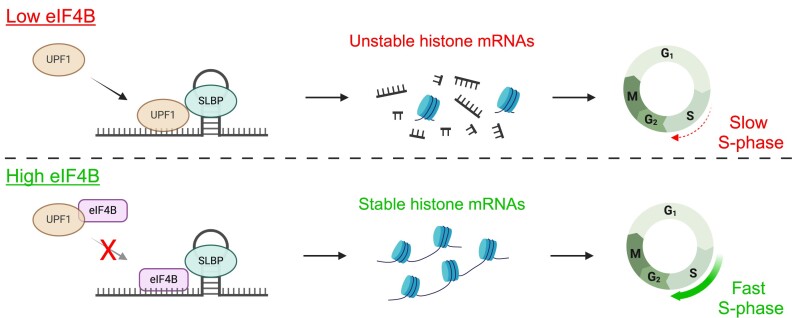

## Introduction

Post-transcriptional control makes an overwhelming contribution to the global regulation of gene expression and this is mediated by RNA binding proteins (RBPs). RBPs regulate all stages of messenger RNA (mRNA) fate, including localization, translation and decay (1), and are capable of recognizing RNA damage (2). The trimeric RBP complex, eukaryotic initiation factor 4F (eIF4F), regulates mRNA translation initiation (3) and is comprised of (i) eIF4E, the cap binding protein, (ii) eIF4G, a large scaffold protein, which has intrinsic G-quadruplex binding activity (4), and (iii) eIF4A, a DEAD-box helicase that binds to and unwinds regions of RNA structure that would otherwise impede the scanning ribosome (3). The dependence of individual mRNAs on eIF4A is specifically dictated by the location of RNA structural elements within their 5′ untranslated regions (UTRs), with increased structure just upstream of the coding sequence (CDS), which is overcome by eIF4A (5,6). The activity of eIF4A is stimulated by the co-factors eIF4B and eIF4H (7–9). In mammalian systems, eIF4B has also been shown to interact with poly(A) binding protein (PABP), which contributes to the stability of the initiation complex (10).

Numerous studies have shown that overexpression of RBPs, including those required for mRNA translation initiation, is associated with tumorigenesis (3,11,12) and the majority of tumours have an addiction to, and dependence on, this process (13–17). As a consequence, there is considerable interest in direct pharmacological targeting of RBPs required for mRNA translation, with several drugs in clinical development (18). There has been a focus on drugs that target eIF4A (18) since net increases in eIF4A activity are frequently associated with worse patient outcomes (19). For example, both eIF4A1 and eIF4B were identified as independent predictors of poor outcome in estrogen receptor (ER)-negative breast cancer, whereas the eIF4A1 inhibitor, PDCD4, was associated with better outcome in ER-positive disease (19). Moreover, in diffuse large B-cell lymphoma (DLBCL), an aggressive form of non-Hodgkin lymphoma, high levels of eIF4B increase the expression of key survival proteins, including BCL-2, DAXX and ERCC5, and are prognostic of poor patient outcome (11).

Signalling from a wide range of upstream signals also controls eIF4B expression and activity. Thus, the transcript that encodes eIF4B contains a 5′ terminal oligopyrimidine-rich region (TOP motif) and expression of eIF4B protein is controlled by signalling through mammalian target of rapamycin (mTOR) (11). In addition, eIF4B is activated by phosphorylation with several oncogenic signalling pathways converging on eIF4B to control its activity, including mechanistic target of rapamycin/phosphoinositide 3-kinases (mTORC1/PI3K), mitogen-activated protein kinases (MAPK) and maternal and embryonic leucine zipper kinase (MELK) (20–23). Although eIF4B has been shown to promote cell proliferation and tumorigenesis (11,24), other than stimulating eIF4A, there is very little mechanistic understanding of eIF4B in this process.

To uncover the full functions of eIF4B in post-transcriptional control of gene expression, we carried out protein interactome analysis of eIF4B in DLBCL in parallel with individual-nucleotide resolution UV cross-linking and immunoprecipitation (iCLIP) analysis. We identify several novel eIF4B interacting proteins, including UPF1 (up-frameshift protein 1), a helicase that plays a key role in the turnover of histone mRNAs at the end of S phase. We show that eIF4B unexpectedly binds the entire length of mRNA transcripts in a mutually exclusive, position-dependent manner, including specific binding to replication-dependent histone mRNAs. Moreover, decreased expression of eIF4B alters histone turnover and delays cell cycle progression. We therefore propose that eIF4B assists the regulation of histone mRNAs during the cell cycle and that this drives proliferation, particularly in tumorigenesis.

## Materials and methods

### Cell culture and siRNA transfection

DoHH-2 (DLBCL cell line) and GM01953 (control B-cell line derived from disease-free individuals) were cultured in RPMI 1640 supplemented with 15% fetal bovine serum (FBS) and 5 mM l-glutamine. HeLa cells were cultured in Dulbecco’s modified Eagle medium supplemented with 10% FBS. DoHH-2 (5 × 10^6^) cells were transfected with 100 nM small interfering RNA (siRNA) using Amaxa 4D Nucleofector (Lonza). Transfections were carried out according to the manufacturer’s instructions using Nucleofection Solution Kit L (VCA-1005) and programme CA-137. HeLa cells were transfected with 30 nM siRNA using Lipofectamine RNAiMAX reagent (Invitrogen) according to the manufacturer’s guidelines for 48 h. ON-TARGETplus siRNA targeting eIF4B (L-020179-00) and non-targeting control siRNA (D-001810-01) were purchased from Horizon Discovery Ltd.

### Cell synchronization and cell cycle analysis

Cells were synchronized to the G1/S-phase boundary using a double thymidine block. After 24-h siRNA transfections, cells were treated with thymidine (2 mM) for 18 h. Thymidine was removed and cell washed three times with pre-warmed media before the addition of deoxycytidine (25 μM) for 8 h. Deoxycytidine was then removed, and cells washed three times in pre-warmed media before the addition of thymidine (2 mM) for a further 16 h. Cells were released from arrest at the G1/S-phase boundary by the addition of deoxycytidine (25 μM). For cell cycle analysis, cells were collected and fixed in 70% ice-cold ethanol. DNA content was determined by the addition of FxCycle Violet stain (4′,6-diamidino-2-phenylindole dihydrochloride) (Thermo Fisher Scientific), which binds double-stranded DNA, for 16 h at 4°C. Incorporation of FxCycle Violet stain was detected using a BD LSRFortessa (BD Biosciences) flow cytometer with a 405-nm laser and a total of 10 000 events were acquired for each sample. All data were analysed using FlowJo data analysis software version 10.1 (FlowJo LLC, USA) and cell cycle state was determined using Watson’s (pragmatic) modelling (25).

### Cell proliferation analysis

Cell proliferation was quantified using the xCELLigence real-time cell analysis DP instrument (Agilent) over 72 h. HeLa cells were transfected with siRNA for eIF4B for 48 h and 5000 cells were then seeded into each well of a microplate E-Plate 16 (Agilent), which contain microelectrode sensors on their surface. As cells proliferate, they generate impedance measurements that enable label-free quantification of cell proliferation in real time. Cell index is an arbitrary value generated by the platform that correlates with cell number.

### Immunoprecipitation

30 × 10^6^ DoHH-2 and GM01953 cells were collected and washed twice in phosphate-buffered saline (PBS). For whole-cell lysis, cells were resuspended in whole-cell lysis buffer [20 mM Tris–HCl, pH 8.0, 137 mM NaCl, 1% IGEPAL CA-630, 2 mM ethylenediaminetetraacetic acid (EDTA), 2 mM MgCl_2_]. For cytoplasmic enriched lysates, cells were resuspended in cell lysis buffer (20 mM Tris–HCl, pH 8, 10 mM NaCl, 3 mM MgCl_2_, 0.35 M sucrose, 0.5% IGEPAL CA-630), incubated on ice for 5 min and the supernatant taken as the enriched cytoplasmic lysate. Insoluble fractions were removed by centrifugation at 10 000 rpm for 10 min at 4°C and protein concentration determined using Pierce BCA Protein Assay Kit (Thermo Fisher Scientific). Dynabeads were covalently coupled with antibodies specific to eIF4B (Bio-Techne, NB100-93308), eIF4A3 (Abcam, ab180573), UPF1 C-terminal (Abcam, ab109353), UPF1 N-terminal (Thermo, MA5-34781) or rabbit immunoglobulin G (IgG) using Dynabeads Antibody Coupling Kit (Life Technologies) according to the manufacturer’s protocol.

Nine hundred micrograms of cleared supernatants were incubated with 1.5 mg antibody-coupled beads for 2 h while rotating at 4°C. To isolate eIF4B interacting proteins, RNA was digested with RNase T1 (10 U per 300 μg protein) to ensure that only direct protein–protein interactions were recovered. Dynabeads were subsequently washed three times in whole-cell lysis buffer and bound proteins were eluted directly into SDS–PAGE (sodium dodecyl sulfate–polyacrylamide gel electrophoresis) loading buffer for analysis by western blotting or mass spectrometry. To isolate RNA bound to eIF4B [RNA immunoprecipitation–quantitative polymerase chain reaction (RIP–qPCR)], cells were harvested in RIP lysis buffer (50 mM Tris–HCl, pH 7.4, 100 mM NaCl, 1% IGEPAL CA-630, 0.1% SDS and 0.5% sodium deoxycholate, supplemented with 1× cOmplete Mini EDTA-free Protease Inhibitor Cocktail and 10 U/ml RNasin). Following immunoprecipitation, beads were washed in RIP lysis buffer and protein was digested using Proteinase K. RNA was then extracted using phenol–chloroform–isoamyl alcohol (25:24:1, v/v). Complementary DNA (cDNA) was synthesized using SuperScript III reverse transcriptase (Thermo Fisher Scientific) and subjected to qPCR analysis. Threshold cycle (CT) values of immunoprecipitation samples were normalized to input RNA (taken prior to immunoprecipitation) and are shown relative to the IgG control.

### Mass spectrometry analysis

Isolated proteins bound to eIF4B were separated on SDS–PAGE gels and serially sectioned prior to digestion with trypsin (26) and liquid chromatography–tandem mass spectrometry was used to identify proteins bound to eIF4B. Extracted tryptic peptides were analysed using data-dependent acquisition on a nanoAcquity UPLC system coupled to a Waters Synapt G2-S HDMS mass spectrometer. The ISOQUANT ‘TOP 3’ method was used for the quantification of proteins. A paired Student’s *t*-test was used to select candidate proteins bound to eIF4B and gene ontology (GO) analysis [http://www.geneontology.org (27,28)] was used to determine enriched biological processes of eIF4B interacting proteins. STRING analysis (version 11, https://string-db.org/) (29) was used to determine characterized interactions between those proteins identified to interact with eIF4B.

### Individual-nucleotide resolution UV cross-linking and immunoprecipitation

iCLIP was performed on DoHH-2 and GM01953 cells. 40 × 10^6^ cells were collected and washed twice in PBS prior to plating on a 10 cm^2^ plate in 6 ml total PBS, to ensure a single-layer cell suspension. Cells were cross-linked using 150 mJ/cm^2^ UVC, resuspended in iCLIP lysis buffer (50 mM Tris–HCl, pH 7.4, 100 mM NaCl, 1% IGEPAL CA-630, 0.1% SDS and 0.5% sodium deoxycholate) supplemented with 1× cOmplete Mini EDTA-free Protease Inhibitor Cocktail and 10 U/ml RNasin (Promega). RNA was partially digested with RNase I (0.6 U) and Turbo DNase (4 U), incubating at 37°C for 3 min and on ice for 3 min. Ten micrograms of eIF4B antibody (Novus, no. NB100-93308) was coupled to Protein G magnetic beads (Life Technologies) and used to immunoprecipitate eIF4B–RNA complexes by rotating at 4°C for 16 h. Beads were washed twice in high-salt buffer (50 mM Tris–HCl, pH 7.4, 1 M NaCl, 1 mM EDTA, 1% IGEPAL CA-630, 0.1% SDS and 0.5% sodium deoxycholate) and twice in PNK wash buffer (20 mM Tris–HCl, pH 7.4, 10 mM MgCl_2_ and 0.2% Tween 20). One-tenth of each sample was labelled with [γ-^32^P]-ATP using PNK and an RNA linker was ligated to the remaining sample. After washing, protein–RNA complexes were eluted directly into 2× NuPAGE loading buffer (Life Technologies) and separated by SDS–PAGE. Protein–RNA complexes were transferred to a nitrocellulose membrane and visualized by autoradiography. RNA complexed to eIF4B had a molecular weight above 80 kDa and RNA was isolated from these fragments using Proteinase K (Roche, 3115879) in PK buffer (100 mM Tris–HCl, pH 7.5, 50 mM NaCl and 10 mM EDTA) by incubating for 20 min at 37°C. Urea (7 M) was added for a further 20 min and RNA was isolated by using phenol–chloroform separation. RNA was reverse transcribed using SuperScript III reagent and Rt1clip, Rt2clip, Rt3clip, Rt4clip, Rt6clip, Rt7clip, Rt8clip, Rt9clip, Rt11clip, Rt12clip, Rt13clip, Rt14clip, Rt15clip and Rt16clip primers, depending on the sample (Supplementary Table S1). Synthesized cDNA was purified using a 6% Tris-Borate-EDTA (TBE)–urea gel and circularized using CircLigase II. cDNA was amplified by PCR using P5/P3 Solexa primers and libraries were then sequenced 1 × 50 using a MiSeq (Illumina) at the Department of Biochemistry of the University of Cambridge.

### iCLIP analysis

For total RNA sequencing, unique molecular identifiers were used to identify and remove PCR duplicates using the clumpify.sh function from the BBMap package (B. Bushnell, sourceforge.net/projects/bbmap/). Trimmed reads were mapped to ENSEMBL GRCh38 using HiSat2 (30), and uniquely mapped reads processed with featureCounts (31) to get a count per gene. Comparison of mRNA levels between the two cell lines was done using the DESeq2 Wald test (32).

For iCLIP, PCR duplicates were removed using the dedupe.sh function from the BBMap package. Then, different experiments were demultiplexed using Je demultiplex (33) before removing experimental barcodes and adapters. Biological replicates with low read numbers were merged into four (for DoHH-2) or two (for GM01953) independent replicates. Trimmed reads were mapped to ENSEMBL GRCh38 using HiSat2. The iCount suite was used to determine cross-link sites, identify peaks of binding and cluster them into binding regions. Then, the number of reads included in peaks for each gene was calculated and compared with those obtained by total mRNA sequencing. The Wald test from the DESeq2 package was used to get a list of genes with significant enrichment (fold change >2, adjusted *P*-value <0.05) in iCLIP compared to total RNA in both cell lines. The sequencing results for all replicates for each condition were then pooled together and re-analysed in the same way using HiSat2 and iCount. Those unique lists of binding regions for each condition were restricted to coding genes enriched in iCLIP and exhibiting >20 reads and used for further analysis.

For metagene analysis and separation of the peaks depending on their binding site on mRNAs, genomic coordinates were converted into transcript coordinates and then related to start and stop codons using a custom script. For simplification, only the longest coding RefSeq mRNA was kept for each gene. Five binding regions were defined for each mRNA: 5′ UTR (>50 nt upstream of start codon), start (50 nt on each side of start codon), CDS (50 nt downstream of start codon to 50 nt upstream of stop codon), stop (50 nt on each side of stop codon) and 3′ UTR (>50 nt downstream of stop codon), and for each region the count was converted into Reads Per Kilobase per Million mapped reads (RPKM). Unsupervised clustering of the genes based on occupancy of those five regions was performed with Multi-experiment Viewer (34) using KMC support, Pearson correlation, distance based on median, five clusters per round, 200 rounds and 70% occurrence in the same clusters.

For motif search, adjacent peaks in genes with significant eIF4B binding were merged into regions. Only regions with >20 reads were considered, and then for each gene we kept only the region with the highest number of peaks. For background, we used regions with >20 reads from a list of genes not showing eIF4B binding [log_2_(fold change) <0.2 and *P*-value >0.1 by the Wald test]. Motif search was performed using HOMER (35) in RNA mode, 20 nucleotides around centre of region and a motif size of 4, 6, 8 and 10 nucleotides.

GO term analysis was performed using the R clusterProfiler package (36), excluding groups of <2000 or >10 genes and with a false discovery rate threshold of 0.001. The GO term profile was then simplified by removing terms with >0.4 similarity with a more significant term.

### Comparison of iCLIP and eCLIP binding regions

Binding peaks from DoHH-2 and GM01953 eIF4B iCLIP datasets were compared to merged clusters of single nucleotide position peaks from UPF1 eCLIP (enhanced CLIP) datasets for K562 and HepG2 (37). The GAT software package (https://gat.readthedocs.io/en/latest/contents.html) was used to test whether the overlap between the predicted UPF1 binding regions and eIF4B binding peaks differed from randomly expected.

### Fluorescence-based RNA binding assay

For RNA binding studies, 40 nM FAM-labelled RNAs (Integrated DNA Technologies) were incubated with increasing concentrations of recombinant eIF4B (8 nM to 2 μM) or UPF1 (0.8–200 nM) in assay buffer (20 mM 4-(2-Hydroxyethyl)piperazine-1-ethane-sulfonic acid (HEPES)/KOH, pH 7.5, 100 mM KCl, 1 mM Tris(2-carboxyethyl)phosphine (TCEP), 2 mM ATP, 2 mM MgCl_2_) in 10 or 20 μl reactions for 60 min at 25°C. For binding reactions in the presence of auxiliary factors, 2 μM eIF4A1, 200 nM UPF1 (TP308018, Sino Biological) or 200 nM stem–loop binding protein (SLBP; TP302361, Sino Biological) were pre-incubated with the RNA in assay buffer for 10 min before eIF4B was added. For UPF1 binding, 2 μM eIF4B was pre-incubated for 10 min with the RNA in assay buffer before UPF1 was added. FAM fluorescence polarization was measured using Spark (Tecan). Dissociation constants were obtained from fitting the experimental data to a one-site binding model using Prism GraphPad 9. Recombinant proteins were generated as described previously (38). Comparisons of the binding affinities were examined for statistical significance by one-way analysis of variance (ANOVA) using GraphPad Prism version 9.3.1 (GraphPad Software, San Diego, CA, USA).

### Fluorescence-based RNA unwinding assay

The His4-FL sequence was modified to contain an internal Cy3 label and a 3′-BHQ (Integrated DNA Technologies) to allow measuring of Histone-SL unwinding by monitoring dequenching of Cy3. Fifty nanomolar RNA substrate was pre-incubated with UPF1 in assay buffer in the absence of ATP for 30 min, before the unwinding reaction was initiated by the addition of 2 mM ATP. When the effect of auxiliary proteins was examined, these were first incubated with the RNA substrate in the absence of UPF1 for 15 min, before the reaction was further pre-incubated together with UPF1. A linear regression model was fitted to the linear part of the progress curve from which the velocity of unwinding was obtained as the slope.

### RNA interactome capture

Cytoplasmic RNA interactome capture (RIC) (2) was carried out on ∼100 × 10^6^ DoHH-2 and GM01953 cells. Cells were collected and washed twice in PBS prior to plating on a 10 cm^2^ plate in 6 ml total PBS, to ensure a single-layer cell suspension. Cells were cross-linked using 150 mJ/cm^2^ UVC and resuspended in cytoplasmic lysis buffer [10 mM HEPES, pH 7.5, 10 mM NaCl, 0.35 M sucrose, 3 mM MgCl_2_, 0.5% NP-40, cOmplete protease inhibitor (Roche), RNasin (Promega)]. After incubation on ice for 3 min, nuclei were pelleted at 1300 × *g* for 5 min at 4°C. Cytoplasmic supernatant was isolated and supplemented with lithium chloride (0.5 M), lithium dodecyl sulfate (0.75%) and 1,4-Dithiothreitol (DTT) (5 mM). Lysates were incubated with oligo(dT)_25_ magnetic beads for 1 h. Beads were subsequently washed once with 20 mM Tris (pH 7.4), 500 mM LiCl, 0.5% LiDS, 1 mM EDTA and 5 mM DTT; twice with 20 mM Tris (pH 7.4), 500 mM LiCl, 0.1% LiDS, 1 mM EDTA and 5 mM DTT for 5 min; twice with 20 mM Tris (pH 7.4), 500 mM LiCl and 1 mM EDTA; and twice with 20 mM Tris (pH 7.4), 200 mM LiCl and 1 mM EDTA. RNA was eluted in 20 mM Tris (pH 7.4) and 1 mM EDTA by incubating beads at 70°C for 3 min. RNA was digested with 125 U Benzonase (Sigma), 40 mg/ml RNase A and 100 U/ml RNase T1 (Thermo Scientific) for 2 h at 37°C. Samples were then diluted in SDS–PAGE loading buffer (50 mM Tris, pH 6.8, 2% SDS, 10% glycerol, 0.1% bromophenol blue and 50 mM DTT) and analysed by western blotting.

### Western blot analysis

Whole-cell extracts were prepared using RIPA buffer (50 mM Tris, pH 7.5, 150 mM sodium chloride, 1% Triton X-100, 0.1% SDS, 0.5% sodium deoxycholate) supplemented with phosphatase inhibitors (PhosSTOP Phosphatase Inhibitor Cocktail; Roche) and protease inhibitors (cOmplete Protease Inhibitor Cocktail; Roche). Protein concentration was quantified using Pierce BCA Protein Assay Kit (Thermo Fisher Scientific). Lysates were diluted in SDS–PAGE loading buffer (50 mM Tris, pH 6.8, 2% SDS, 10% glycerol, 0.1% bromophenol blue and 50 mM DTT) and equal amounts of proteins were subjected to SDS–PAGE and western blot analysis. Primary antibodies used were eIF4B (CST, no. 3592), Daxx (Millipore, no. 07-471), PABP (CST, no. 4992), eIF4A1 (Abcam, no. ab31217), eIF4A2 (Abcam, no. ab31218), eIF4A3 (Santa Cruz, no. sc-365549), eIF4G (CST, no. 2498), DDX3 (CST, no. 8192), DDX6 (Abcam, no. ab70455), G3BP1 (Abcam, no. ab56574), BCL2 (CST, no. 2872), c-Myc (Epitomics, no. 1472), DAXX (Upstate, no. 07-471), GAPDH (CST, no. 2118), DDX3 (Abcam, no. 37160), FMR1 (CST, no. 4317S), UPF1 (CST, no. 12040), pUPF1 (CST, no. 84283), Cyclin A2 (CST, no. 4656), Cyclin B1 (CST, no. 4138), Cyclin E2 (CST, no. 4132), Vinculin (Sigma, no. V9131), Nucleolin (GeneTex, no. GTX13541), PTB [BB7 clone, home-made (39)] and β-tubulin (CST, no. 2146). For enhanced chemiluminescence detections, the secondary antibodies used were horseradish peroxidase-conjugated α-mouse (Dako, no. P0447) or α-rabbit (GE Healthcare, no. NA934V). For fluorescent detection using LI-COR Odyssey imaging systems (LI-COR Biosciences), the secondary antibodies used were IRDye Light-labelled α-mouse (CST, no. 5257 and no. 5470) and α-rabbit (CST, no. 5366 and no. 5151).

### RNA stabilization assay

Cells transfected with siRNAs were seeded into six-well plates 24 h prior to treatment. Cells were treated with the transcriptional inhibitor flavopiridol (1 μM) for the indicated time and cell lysates were collected in the TRIzol reagent. Total RNA was isolated according to the manufacturer’s protocol. Briefly, 200 μl chloroform was added to 1 ml TRIzol and samples were centrifuged at 12 000 × *g* for 15 min at 4°C. RNA was precipitated from the upper aqueous phase using isopropanol and resuspended in water. cDNA was reverse transcribed from 1–2 μg purified total RNA using SuperScript III reverse transcriptase (Thermo Fisher Scientific) and subjected to qPCR analysis. mRNA levels were determined relative to the 0-h time point and mRNA half-life was calculated using a one-phase decay curve with GraphPad Prism version 9.3.1 (GraphPad Software, San Diego, CA, USA).

### qPCR analysis

cDNA was amplified in real time using SensiFAST SYBR^®^ Lo-ROX reagent (Roche) according to the manufacturer’s instructions. All qPCR reactions were performed using the QuantStudio 6 Flex real-time PCR system (Thermo Fisher Scientific). Primer sequences used were GAPDH (forward: AATCCCATCACCATCTTCCA; reverse: TGGACTCCACGACGTACTCA), Actin B (forward: CTTAGTTGCGTTACACC; reverse: ATTGTGAACTTTGGGGG); Histone H4C8 mRNA (forward: AAGGTTTGGGTAAGGGAGGA; reverse: TTTGGCGTGCTCTGTGTAAG), Histone H3C10 mRNA (forward: CTATCGGCCTGGTACAGTGG; reverse: GGTTGGTGTCCTCAAAGAGC), Histone H1-4 mRNA (forward: TTCAACATGTCCGAGACTGC; reverse: AGGCGGCAACAGCTTTAGTA), Histone H2AC6 mRNA (forward: CGCTGGTTTTGGTGATTTTT; reverse: CTCGGCGGTCAGGTACTCTA), Histone H2BC15 (forward: TAAGCGGTAGGTTGACAGAGC; reverse: GGCTCACTTGGAACTGGTGT), Histone H4C3 (forward: CGAGACGCCGTCACCTATAC; reverse: CCCCTGACGTTTTAGGGCAT), Histone H2AC13 (forward: AGAAGACTCGCATCATCCCG; reverse: TCCAGGCTTCTACTTGCCCT) and Histone H2AX (forward: ATCGCCGATTTCGGTCTGG; reverse: TGTGCCTGTTACCAAGTGCT).

## Results

### eIF4B interacts with proteins that have a role in repression of translation and RNA turnover

**Figure 1.**
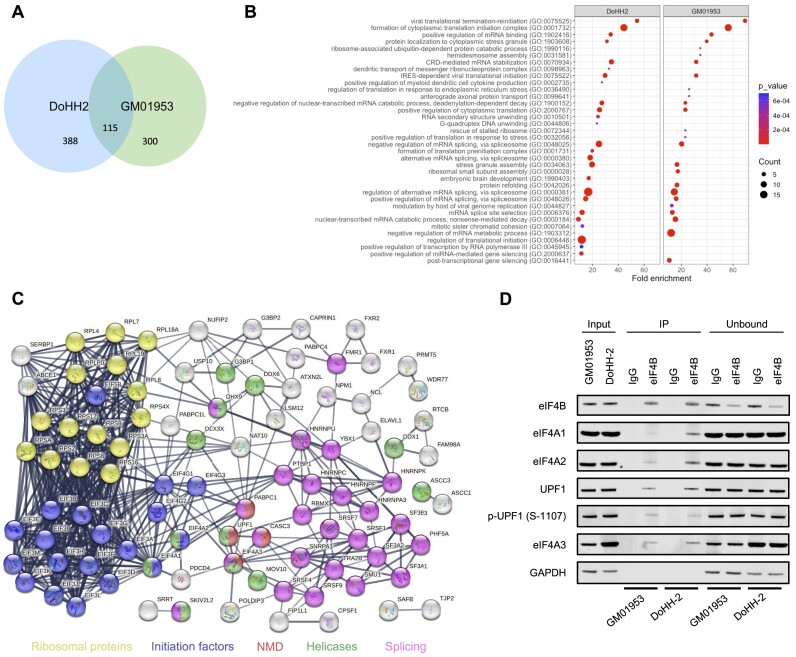
Identification of novel eIF4B interacting proteins involved in nonsense-mediated decay (NMD). (**A**) Venn diagram of eIF4B interacting proteins identified by mass spectrometry in DoHH-2 and GM01953 cells. All co-immunoprecipitations were subjected to RNase digestion, allowing the isolation of direct eIF4B interacting proteins. Proteins were identified as eIF4B binding when showing a significant enrichment (*P*-value <0.05) relative to the IgG control immunoprecipitation. (**B**) GO analysis of the top 25 biological processes associated with eIF4B interacting proteins in DoHH-2 and GM01953 cells. Fold enrichment shows the enrichment of genes above the expected and significance was determined by Fisher’s exact test. (**C**) STRING interaction analysis of the eIF4B interacting proteins conserved between DoHH-2 and GM01953 cells. Proteins are coloured according to cellular function: yellow, ribosomal proteins; blue, initiation factors; red, NMD; green, helicases; pink, splicing factors. (**D**) Cell lysates from DoHH-2 and GM01953 cells were immunoprecipitated for eIF4B or IgG. After RNA digestion and washing to remove RNA-dependent interactions, directly interacting proteins were analysed by immunoblotting with the indicated antibodies. GAPDH was used as a negative control because it did not bind to eIF4B in the mass spectrometry experiments. Blots shown are representative of three independent experiments.

Although eIF4B has been implicated in regulating the growth and proliferation of cancer cells (11,20,22,23), the exact mechanisms are unclear. To gain further insight, first we determined the protein binding partners of eIF4B, which are not well described. We have shown previously that high expression of eIF4B (with no associated increase in eIF4A) is associated with poor outcome in patients with DLBCL (11). We confirmed these data and additionally show that there are no differences in expression of eIF4A2 or the helicase DDX6 between our DLBCL-derived cell line, DoHH-2, and our control disease-free B-cell line, GM01953 (Supplementary Figure S1A). Although not isogenic cell lines, we have previously shown overexpression of eIF4B in DoHH-2 compared to GM01953 (11), providing a suitable model to investigate the role of eIF4B further. To identify additional binding partners of eIF4B and to determine potential differences in eIF4B-containing RBP complexes in cell lines derived from patients with DLBCL, a series of immunoprecipitation experiments were carried out in triplicate. Following digestion with RNase T1, direct eIF4B interacting proteins were identified by mass spectrometry. In total, 115 proteins were identified as direct interactors of eIF4B in both DoHH-2 and GM01953, whereas 388 and 300 interactors were unique to DoHH-2 and GM01953, respectively (Figure 1A and Supplementary Table S2). GO analysis shows that many of these proteins are RBPs likely to be involved in the positive regulation of mRNA translation (Figure 1B).

These include ribosomal proteins, consistent with the role of eIF4B in translation regulation and its direct binding to ribosomal RNA (40), and the DEAD-box RNA helicase DDX3. However, proteins involved in pathways considered to be negative regulators of mRNA translation and stability were also identified, e.g. those required for stress granule/body formation such as G3BP1, FMRI and the related FXR1 (41), the DEAD-box RNA helicases DDX6 and eIF4A2, and NMD and RNA turnover regulatory proteins, including UPF1 and eIF4A3 (Figure 1B and C, and Supplementary Table S2). As G3PB1 is a known component of stress granules, we compared our eIF4B interactome to core stress granule proteins (42). Interestingly, 14 of the 115 eIF4B interacting proteins are also core components of stress granules (Supplementary Figure S1B), highlighting a possible underappreciated role of eIF4B in stress granule biology.

These novel interactions with eIF4B were confirmed by immunoprecipitation and western blotting (Figure 1D and Supplementary Figure S1C). In some cases, increased binding of eIF4B in DoHH-2 cells could be attributed to increased expression, e.g. FMR1, eIF4A3 and DDX3, but this was not the case for other proteins, including UPF1, eIF4A1, eIF4A2 and G3BP1. Some interactions may also be linked to the increased rates of translation initiation in our tumour cell line. For example, binding of eIF4B to eIF4A1 enhances translation initiation, and we observe an increase of this interaction in DoHH-2 (tumour) relative to GM01953 (non-tumour). Furthermore, eIF4B was shown to bind phosphorylated UPF1 in both cell lines (Figure 1D), suggesting that this interaction may be more general and could be linked to UPF1 activity.

To confirm the interaction between eIF4B and two of the proteins implicated in regulation of NMD, eIF4A3 and UPF1, we carried out the inverse immunoprecipitations. We observed that eIF4B co-immunoprecipitated with eIF4A3 from DoHH-2 whole-cell lysates (Supplementary Figure S1D). As hyperphosphorylated UPF1 has previously been shown to accumulate in the cytoplasm (43), we generated cytoplasmic enriched DoHH-2 lysates and immunoprecipitated UPF1 with antibodies that recognized either the C-terminus or N-terminus. As an additional control, we also immunoprecipitated eIF4B. Importantly, following immunoprecipitation of UPF1 and digestion of all RNA, we observed binding of eIF4B, thus confirming the direct interaction between UPF1 and eIF4B (Supplementary Figure S1E).

Taken together, these data suggest a putative new role for eIF4B in the negative regulation of protein synthesis and mRNA stability.

### eIF4B binds to defined sites along the entire length of mRNA transcripts

**Figure 2.**
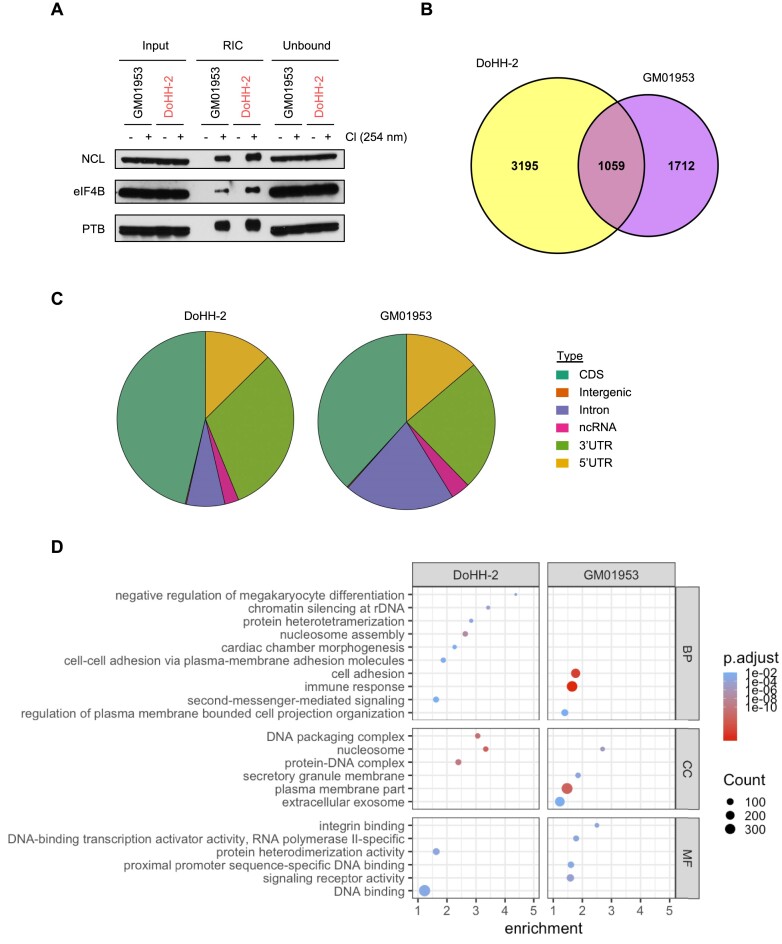
Identification of eIF4B RNA binding sites by iCLIP in DLBCL. (**A**) Cell lysates from GM01953 and DoHH-2 were subjected to RIC after UVC (254 nM) cross-linking (Cl). RIC samples were analysed by immunoblotting for the indicated RBPs and loading was normalized to RNA. Blots shown are representative of three independent experiments. (**B**) Venn diagram of the number of significant (Wald test *P*-value <0.05) individual RNAs bound by eIF4B in DoHH-2, GM01953 or both cell lines. (**C**) Distribution of iCLIP peaks across RNA species bound by eIF4B in DoHH-2 and GM01953 cells. (**D**) GO analysis of mRNAs bound by eIF4B in DoHH-2 and GM01953 cells (BP, biological processes; CC, cellular components; MF, molecular functions). Fold enrichment shows the enrichment of genes above the expected and significance was determined by Fisher’s exact test.

To determine whether the increased level of eIF4B in DLBCL was associated with additional RNA binding capacity, RIC was carried out (Figure 2A). RIC utilizes oligo(dT) beads to capture RBPs bound to cellular RNA after UV cross-linking and demonstrated that eIF4B binds an increased amount of RNA in DoHH-2 cells compared to GM01953. To assess the target sites of eIF4B in DoHH-2 compared to GM01953, iCLIP analysis was carried out. RNA was cross-linked to interacting proteins by UV and eIF4B-bound RNA was determined by next-generation sequencing. In total, we identified 3195 and 1712 individual RNAs bound by eIF4B in DoHH-2 and GM01953, respectively, whereas 1059 were bound in both cell lines (Figure 2B). Moreover, we observed ∼10-fold more binding sites in DoHH-2 (253 791 binding sites) compared to GM01953 (27 807 binding sites).

The majority of interactions with eIF4B were identified within mRNA transcripts, whereas only 3.5% (GM01953) and 2.6% (DoHH-2) of binding occurred within non-coding RNA, such as rRNA [in agreement with the data obtained in yeast (40)] (Figure 2C and Supplementary Table S3). The distribution of eIF4B binding sites across mRNAs showed the expected binding at the 5′ UTR, consistent with a role for eIF4B in stimulating the activity of eIF4A during translation initiation; however, binding of eIF4B was observed across the entire length of mRNA transcripts (Figure 2C and Supplementary Figure S2A).

There were also differences observed between DoHH-2 and GM01953 in terms of distribution of binding as eIF4B bound more sites within the CDS and 3′ UTR of mRNAs in DoHH-2 (Figure 2C). GO analysis was used to identify the biological processes, cellular components and molecular functions of the mRNAs bound by eIF4B. Interestingly, distinct differences in mRNA binding were observed in both cell lines, most notably in GO terms relating to DNA packaging, nucleosome and nucleosome assembly (Figure 2D).

### eIF4B displays distinct binding patterns on different subsets of transcripts

**Figure 3.**
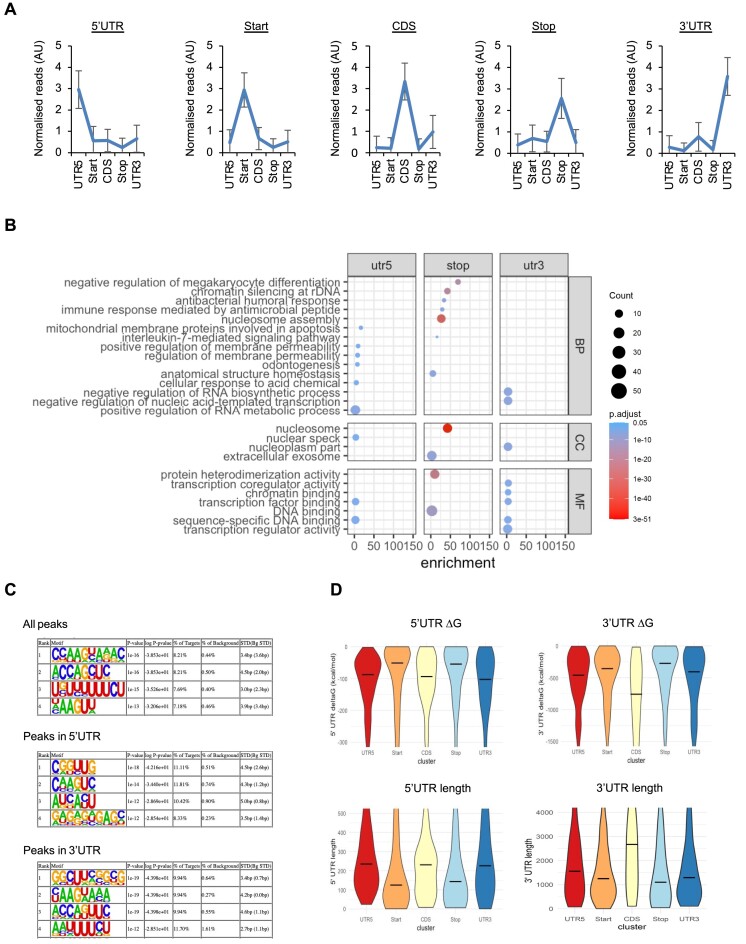
eIF4B binds across the entire length of mRNA transcripts. (**A**) A subset of mRNAs bound by eIF4B in DoHH-2 cells were classified within five distinct groups based on the location of eIF4B binding (5′ UTR = 164 genes; start codon = 199 genes; CDS = 56 genes; stop codon = 125 genes; 3′ UTR = 124 genes). Data values show mean normalized reads with standard deviation (*n* = 4). (**B**) GO analysis of the RNAs bound by eIF4B at the 5′ UTR, stop codon and 3′ UTR (BP, biological processes; CC, cellular components; MF, molecular functions). Fold enrichment shows the enrichment of genes above the expected and significance was determined by Fisher’s exact test. (**C**) eIF4B binding motifs were analysed within all peaks, 5′ UTR and 3′ UTR eIF4B binding groups shown in panel (A). Motifs are ranked by enrichment *P*-value calculated by HOMER (35). % of targets: number of target sequences with motif/total targets; % of background: number of background sequences with motif/total background; STD(Bg STD): standard deviation of position in target and background sequences. (**D**) The length and Δ*G* of 5′ and 3′ UTRs of mRNAs within each eIF4B binding cluster (shown in panel A) were analysed. mRNAs are grouped by the region they are bound by eIF4B (5′ UTR, start codon, CDS, stop codon, 3′ UTR).

The transcripts bound by eIF4B in DoHH-2 cells can be clustered based on the region of binding (Figure 3A) and the data suggest that the location of binding on the mRNA is mutually exclusive in a small subset of transcripts. For example, transcripts showing an enrichment of eIF4B binding to the 5′ UTR do not show enrichment in binding elsewhere on the transcript (Figure 3A and Supplementary Figure S3A). In general, eIF4B binding within the 5′ UTR of mRNAs is associated with protein products that function in cell growth and proliferation (Supplementary Figure S3A), whereas binding near the stop codon shows enrichment for mRNAs that encode histones (i.e. nucleosomes) (Figure 3B and Supplementary Figure S3A), suggesting that binding location may be associated with the function of the encoded proteins. Since eIF4B interacts with a range of RBPs (Figure 1), one hypothesis, consistent with these data, is that eIF4B could differentially regulate subsets of mRNAs depending on its interacting protein partners, resulting in translational activation of some mRNAs and repression/turnover of others.

The eIF4B binding motifs of the transcripts within each cluster were analysed, and again, these show distinct region-specific differences (Figure 3C). Interestingly, one binding motif in the 5′ UTR (GAGAGAGAGC, Figure 3C) is similar to that reported for eIF4A (38,44), consistent with the role of eIF4B in stimulating eIF4A. To examine whether there are RNA structural differences related to the position-dependent eIF4B binding, we plotted the Δ*G*, 5′ UTR and 3′ UTR length for each eIF4B binding cluster. The 5′ UTR length of mRNAs bound by eIF4B in the CDS, 5′ UTR and 3′ UTR binding clusters is overall longer and predicted to be more structured (Figure 3D). Conversely, the 3′ UTR length of mRNAs bound by eIF4B within the CDS cluster is longer and more structured than that in the stop cluster, possibly due to the increased number of histone mRNAs identified within the stop cluster.

Taken together, these data suggest that eIF4B binds different subsets of mRNAs in positions that are mutually exclusive.

### eIF4B binds directly to replication-dependent histone mRNAs

As eIF4B is known to enhance the helicase activity of eIF4A, it was not surprising to identify binding of eIF4B to the 5′ UTR region of mRNAs. However, more unexpectedly, we identified binding of eIF4B near the stop codon of a subset of mRNAs, which included a large enrichment of replication-dependent histone mRNAs. It was therefore important to confirm eIF4B binding within the unexpected ‘stop codon’ cluster.

**Figure 4.**
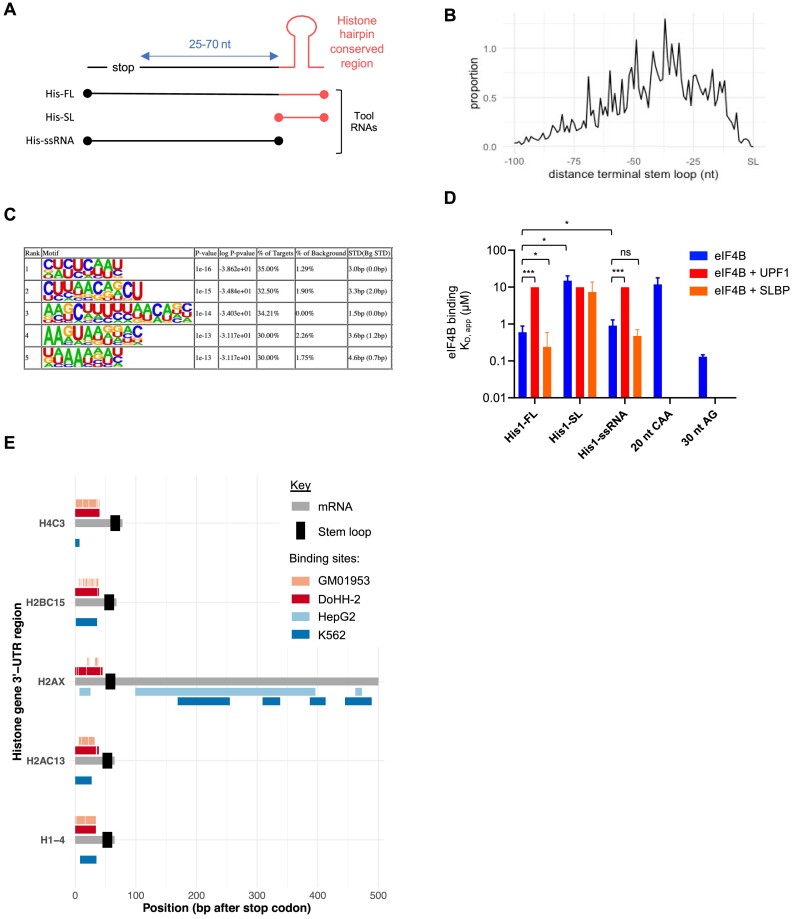
eIF4B binds histone mRNAs. (**A**) Schematic representation of replication-dependent histone 3′ UTRs and tool RNAs generated for fluorescence-based RNA binding assays. (**B**) Distribution of eIF4B binding sites relative to the stem–loop on replication-dependent histone mRNAs from DoHH-2 iCLIP analysis. Proportion is an arbitrary value of eIF4B binding across all histone mRNAs. (**C**) eIF4B binding motifs were analysed within histone mRNAs in DoHH-2 cells. Motifs are ranked by enrichment *P*-value calculated by HOMER (35). % of targets: number of target sequences with motif/total targets; % of background: number of background sequences with motif/total background; STD(Bg STD): standard deviation of position in target and background sequences. (**D**) Fluorescence-based RNA binding assay showing eIF4B binding to tool RNAs based on histone 1 mRNA. Dissociation constants of RNA binding affinity of eIF4B alone and in the presence of UPF1 and SLBP. Error bars represent mean ± standard deviation (*n* = 3 independent experiments); *P ≤ 0.05 and ***P ≤ 0.001 by ANOVA. His1-FL: full-length sequence; His1-SL: containing histone stem–loop only; His1-ssRNA: full-length sequence minus the stem–loop; 20 nt CAA: negative control RNA; 30 nt AG: positive control RNA. Binding to 20 nt CAA and 30 nt AG was only carried out in the presence of eIF4B alone. (**E**) Comparison of eIF4B iCLIP binding sites from DoHH-2 (red) and GM01953 (peach) with UPF1 eCLIP binding sites from K562 (dark blue) and HepG2 (light blue) on histone mRNAs. All mRNAs (grey) were aligned at the stop codon and the stem–loop location is indicated in black.

Replication-dependent histone mRNAs are unique since they contain a stem–loop (a six-base stem and four-nucleotide loop) at their 3′ end instead of a poly(A) tail (45) (Figure 4A), and our iCLIP data show that the distribution of eIF4B binding is upstream of this structure (Figure 4B). Moreover, as eIF4B was bound to many replication-dependent histones, several conserved sequences were identifiable within our dataset (Figure 4C). To examine the interaction of eIF4B with histone mRNAs, a fluorescence-based *in vitro* binding assay was used (38). The histone 1 mRNA sequence was used to design ‘tool’ RNAs that encompassed the stop codon to the stem–loop (Figure 4A and Supplementary Table S4). These include a full-length RNA (His1-FL), one without the stem–loop (His1-ssRNA), the stem–loop alone (His1-SL), a negative control RNA sequence (CAA) and a positive control sequence (30 nt AG), which is an eIF4A-dependent sequence (38,44) and overlaps with the identified eIF4B 5′ UTR motif (Figure 3C). By determining the dissociation constant (*K*_D_), where a lower *K*_D_ indicates increased binding affinity, these data suggest that eIF4B binds to full-length histone 1 (His1-FL; Figure 4D) and, in support of our iCLIP data, preferentially binds to the ssRNA region upstream of the stem–loop (His-ssRNA; Figure 4D). Moreover, eIF4B binding to the ssRNA region was also observed with tool RNAs based on the histone 4 mRNA (Supplementary Figure S4A). As our *in vitro* assay measured binding of recombinant eIF4B to synthetic histone RNAs, it was important to confirm endogenous eIF4B binding to both histone 1 (H1–4) and histone 4 (H4C8) mRNAs. We therefore carried out RIP–qPCR analysis following eIF4B immunoprecipitation from DoHH-2 cells and observed eIF4B binding to both H1–4 and H4C8 mRNAs relative to the IgG control (Supplementary Figure S4B).

UPF1 is an RNA helicase that binds to SLBP and the stem–loop region of histone mRNAs, and is required for rapid histone mRNA degradation at the end of S phase or in response to replication stress (46). As we previously identified a direct interaction between eIF4B and UPF1 (Figure 1), it was important to determine how UPF1 and SLBP affected eIF4B binding in our *in vitro* assay. Interestingly, the presence of UPF1 blocked eIF4B binding to the His1-ssRNA RNA, whereas SLBP had no effect (Figure 4D). As eIF4B is known to promote the helicase of eIF4A, we next wanted to determine whether the interaction between eIF4B and UPF1 could affect UPF1 helicase activity. We used a fluorescence-based RNA unwinding assay using a tool RNA based on histone 4 mRNA to monitor relative unwinding by UPF1 in the presence of eIF4B or SLBP (Supplementary Figure S4C). Although UPF1 showed preference to the histone RNA over our negative control AG (eIF4A1-dependent) sequence, the addition of eIF4B had minimal impact on UPF1 activity. Furthermore, we observed that UPF1 had a much greater affinity for the tool RNAs *in vitro* than eIF4B (Supplementary Figure S4D). Taken together, these *in vitro* data suggest that eIF4B and UPF1 could compete for the same binding sites on histone mRNAs.

To determine whether the binding sites of eIF4B overlapped with those of UPF1, we compared the binding regions from our eIF4B iCLIP data to published UPF1 eCLIP datasets from K562 and HepG2 cells (37). Our comparisons indicated that the binding regions of UPF1 and eIF4B significantly overlap within the 3′ UTRs (Supplementary Table S5). Moreover, an overlap in eIF4B and UPF1 binding upstream of the stem–loop was observed on histone mRNAs between DoHH-2 and K562 (Figure 4E and Supplementary Table S5). Binding of eIF4B upstream of the stem–loop was extensive in our iCLIP analysis (Supplementary Figure S4E), and although we did observe some overlap of UPF1 and eIF4B binding sites in HepG2 and GM01953 (Figure 4E), it was not significant (Supplementary Table S5). However, it is important to note that the lower number of reads from GM01953 and HepG2 limited the statistical power of this analysis.

Taken together, these data suggest that eIF4B binds histone mRNAs upstream of the stem–loop and may compete with UPF1 for binding sites, suggesting that eIF4B could play a role in the either RNA stability or turnover of replication-dependent histone mRNAs.

### eIF4B regulates the stability of replication-dependent histone mRNAs and contributes to S-phase progression

**Figure 5.**
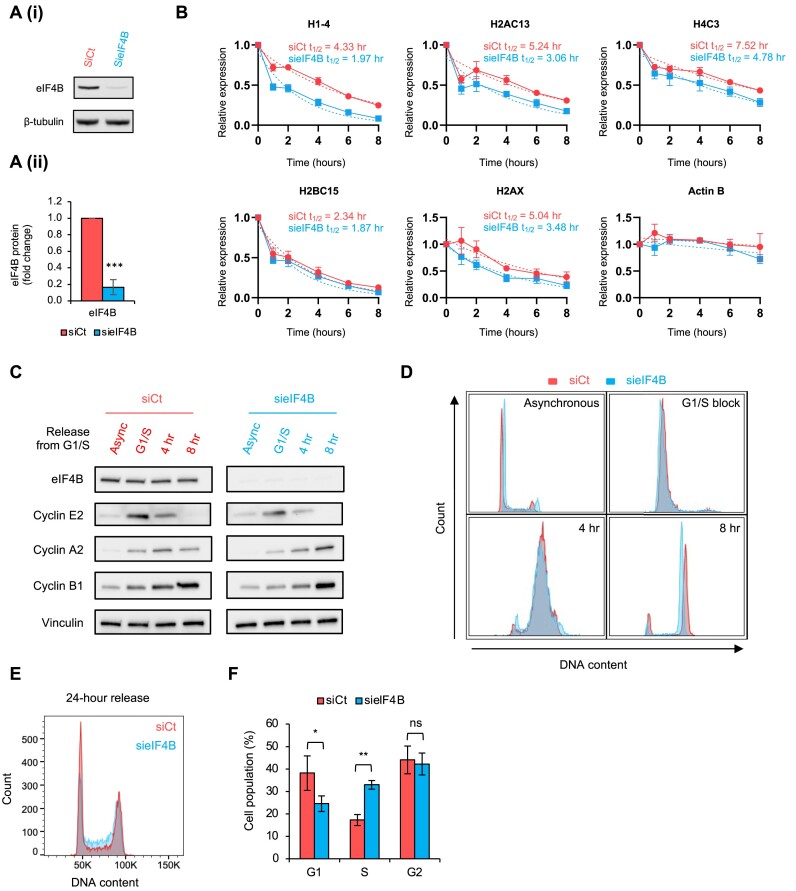
eIF4B stabilizes histone mRNAs and accurate S-phase progression. (**A**) (i) HeLa cells were transfected with a control non-targeting siRNA or an siRNA specific for eIF4B (30 nM) for 72 h. Cells were lysed and knockdown efficiency was determined by immunoblotting with the indicated antibodies. Blots are representative of three independent experiments. (ii) Quantification of eIF4B protein levels from panel (i) normalized to β-tubulin levels. Error bars represent mean ± standard deviation (*n* = 3 independent experiments); ***P ≤ 0.001 by unpaired Student’s *t*-test. (**B**) HeLa cells were transfected with an siRNA specific for eIF4B (30 nM) or a non-targeting control for 72 h and treated with flavopiridol (1 μM) to inhibit transcription for the indicated time. Total RNA was isolated and synthesized cDNA was used to quantify the indicated histone mRNA levels using qPCR. Estimation of mRNA half-life (*t*_1/2_) was determined using a one-phase decay model (dotted line). Actin mRNA was used as a negative control and eIF4B depletion did not reduce actin mRNA half-life within the time frames analysed. Mean values for each time point are shown (solid line) and error bars represent standard deviation (*n* = 3 independent experiments). (**C**) HeLa cells were transfected with an siRNA specific for eIF4B (30 nM) or a non-targeting control and subjected to a double thymidine block to arrest cells at the G1/S checkpoint (G1/S). Cells were released from the block for either 4 or 8 h and immunoblotted with the indicated antibodies to determine the expression of cyclin proteins. Async, asynchronous cells. Blots shown are representative of three independent experiments. (**D**) Cells were treated as in panel (C) and collected and fixed for cell cycle analysis. DNA was stained using FxCycle Violet dye and quantified by flow cytometry. Histograms of stained DNA are representative of three independent experiments. (**E**) HeLa cells were transfected with an siRNA specific for eIF4B (30 nM) or a non-targeting control and subjected to a double thymidine block to arrest cells at the G1/S checkpoint (G1/S). Cells were released from the block for 24 h and fixed for cell cycle analysis. DNA was stained using FxCycle Violet dye and quantified by flow cytometry. Histogram of stained DNA is representative of three independent experiments. (**F**) Quantification of cell cycle stage from panel (E) using Watson’s (pragmatic) model. Error bars represent mean ± standard deviation (*n* = 3 independent experiments); *P ≤ 0.05 and **P ≤ 0.01 by unpaired Student’s *t*-test (ns, not significant).

The consequences of eIF4B binding to its target mRNAs include that it directly controls protein levels by stimulating mRNA translation or that it controls protein expression indirectly through regulating mRNA stability and turnover. Our iCLIP data show that eIF4B binds to the structured 5′ UTR of CDK4 mRNA (Supplementary Figures S3A and S5A), consistent with its role as a stimulator of eIF4A. We validated eIF4B binding to CDK4 mRNA by RIP–qPCR (Supplementary Figure S5B) and show that eIF4B preferentially binds to the region 5′ of the start site using our *in vitro* binding assay with tool CDK4 RNAs (Supplementary Figure S5C and D, and Supplementary Table S4). In addition, we observe an increase in CDK4 protein expression in DoHH-2 cells, but only a modest increase in mRNA levels, suggesting that eIF4B may play some role in the translation of CDK4 mRNA (Supplementary Figure S5E). However, more uniquely, our data suggest a possible role for eIF4B in the regulation of replication-dependent histone mRNAs. It is known that on entering S phase the cellular levels of histone mRNAs increase, and at the end of S phase they are rapidly degraded via a process that requires UPF1 (46). As we identified that eIF4B directly interacts with UPF1 and our analysis shows that they may compete for overlapping binding sites on histone mRNAs, we explored the effect of eIF4B knockdown on histone mRNA levels. It was not possible to obtain robust knockdown of eIF4B in DoHH-2 cells (Supplementary Figure S5F and G). However, given the overlap of UPF1 and eIF4B binding was observed between different cell lines (Figure 4E), we reasoned that if eIF4B had an effect in controlling histone mRNA stability, this was unlikely to be cell line specific. Therefore, HeLa cells were used for the subsequent experiments. EIF4B expression was reduced by siRNA (Figure 5A) and cells were treated with a high concentration of flavopiridol to block transcription (47). Samples were collected over the subsequent 8-h period and reverse transcription qPCR was used to quantify histone mRNA levels. We then used one-phase decay to calculate the half-life of histone mRNAs that showed an overlap in eIF4B and UPF1 binding sites.

Depletion of eIF4B reduced the estimated mRNA half-life of histone H1–4, H2AC13, H4C3 and H2AX compared to a non-targeting control siRNA, with no impact on actin mRNA levels (Figure 5B). Although the impact on H2BC15 was more modest (Figure 5B), these data suggest that eIF4B may stabilize histone mRNAs. To explore this further, we expanded to three other histone mRNAs that were bound by eIF4B in our iCLIP analysis (H2AC6, H4C8 and H3C10) and the data show that depletion of eIF4B generally decreases histone mRNA half-life; however, this was to a slightly lesser extent for H2AC6 and H4C8 (Supplementary Figure S5H).

The efficient regulation of histone mRNA synthesis and turnover is crucial for the rapid and accurate progression through S phase (48). Therefore, to determine the effect of eIF4B on histone mRNA stability and the cell cycle, eIF4B levels were depleted by siRNA and a double thymidine block was used to synchronize cells at the G1/S border. Following release from the arrest, the expression of cyclin proteins was analysed by western blot (Figure 5C). As expected, cyclin E expression was highest after G1/S arrest, and this expression decreased at 4 h as cells progress into S phase. However, although cyclin A2 expression peaked at 4 h in control cells, expression did not peak until 8 h in eIF4B-depleted cells. Furthermore, control cells showed elevated levels of cyclin B from 4 h, indicating entry to mitosis, whereas this was delayed to 8 h in eIF4B-depleted cells, suggesting that S-phase progression was delayed in eIF4B-depleted cells. Fluorescence-activated cell sorting analysis was used to confirm that cell cycle progression was delayed in eIF4B-depleted cells (Figure 5D). The double thymidine block was confirmed to pause cells at the G1/S border (G1/S block) and, following release from the arrest, progression through S phase (4 h release) into G2 (8 h release). Consistent with the analysis of cyclin levels, eIF4B-depleted cells showed delayed progression through the cell cycle, most notably seen by an increase of cells in G2 at 8 h compared to control cells, which were already progressing through to G1 (Figure 5D). Quantification of cells in S phase is challenging without the incorporation of thymidine analogues such as 5-ethynyl 2′-deoxyuridine (EdU). However, as cells should rapidly return to asynchronous populations following release from a G1/S block, we determined the ability eIF4B-depleted cells to do this over 24 h. We used Watson’s pragmatic modelling (25) to determine cell cycle state following DNA staining (Supplementary Figure S5I). Whereas control cells return to a mostly asynchronous population by 24 h, eIF4B-depleted cells do not and increased levels of cells in S phase persist for over 24 h, indicating that eIF4B may assist with efficient progression through S phase (Figure 5E and F). Consistent with these data, acute depletion of eIF4B also subtly reduced HeLa cell proliferation (Supplementary Figure S5J).

Taken together, these data suggest that eIF4B may be required for the regulation of histone mRNA stability and this in turn contributes to the regulation of S-phase progression.

## Discussion

To date, in addition to well-described canonical RBPs, ∼1500 additional proteins have been identified that have the capacity to bind RNA (49–51). Importantly, the data suggest that RBPs do not act singly, but instead a combinatorial code exists whereby complexes comprised of different subsets of RBPs are formed under distinct cellular conditions. Such complexes, by controlling gene expression post-transcriptionally, are essential for the full execution of a wide range of cellular processes (52,53). Here, we show that the RBP, eIF4B, is also multi-functional. Thus, while eIF4B interacts directly with 5′ UTRs of mRNAs, consistent with its role as a stimulator eIF4A, it also binds at distinct sites across mRNA transcripts (Figure 2C). Importantly, binding of eIF4B to subsets of mRNAs is mutually exclusive and position-dependent (Figure 3A). Of particular interest was the interaction of eIF4B with a region upstream of the stem–loop in mRNAs that encode replication-dependent histones.

Metazoan histone mRNA regulation is inherently linked to the cell cycle due to the rapid expression required for S phase (54). As such, histones are expressed from multiple genes, found in histone gene clusters, which produce ∼75 histone mRNAs (55). Histone mRNAs are not polyadenylated, but instead contain a highly conserved stem–loop at their 3′ end (45), which is bound by SLBP (56,57). In fact, histone mRNAs are the only known target of SLBP (58), and SLBP regulates all aspects of histone mRNA metabolism, including stability, translation and degradation (54). As histone mRNAs are capped at their 5' end, once exported to the cytoplasm, SLBP interacts with SLIP1 to enable histone mRNA circularization (59), and to eIF3g (60) to promote mRNA translation. However, a major regulatory step of histone mRNAs is their rapid degradation at the conclusion of S phase (46). The underlying molecular mechanisms that control translation-dependent degradation of histone mRNAs are complex and not fully understood, although it has been shown that remodelling of SLBP/histone RNP complex is required to switch from an actively translating mode to a degradation mode. As part of this process, the interaction between the CBP80/20-dependent translation initiation factor (CTIF) and SLBP, which are required for efficient histone mRNA translation, is disrupted via a competition between CTIF and UPF1 for SLBP binding (61). This is controlled by DNA transcription-dependent phosphorylation of UPF1 by ATR and DNAPK, which increases its interaction with SLBP, promoting release of CTIF and eIF3 from SLBP-containing histone mRNP (61). In parallel, hyperphosphorylated UPF1 recruits PNRC2 and SMG5, triggering decapping followed by 5′-to-3′ degradation of histone mRNAs (61,62). Our data suggest that eIF4B has hitherto unappreciated function in this process, and that decreasing the expression of eIF4B decreases the half-life of mRNA encoding histone-dependent mRNAs (Figure 5B). In support of a direct role for eIF4B in this process, we show that eIF4B interacts directly with a key component of the remodelling complex, UPF1 (Figure 1). Although it is unclear whether eIF4B binding to UPF1 occurs on the histone mRNA or whether it affects the interaction between UPF1 and SLBP, we show that eIF4B interacts with phosphorylated UPF1. Moreover, our *in vitro* data indicate that eIF4B had no effect on UPF1 helicase activity, instead suggesting that eIF4B and UPF1 may compete for the same binding regions on histone mRNAs. To further study the biological relevance of this interaction, it would be useful to determine when this interaction occurs during the cell cycle and to conduct structural analysis to guide the generation of mutant proteins that would disrupt the interaction and study the impact on cell fate.

Interestingly, we identified almost the entire eIF3 complex interacting with eIF4B (Figure 1C). Although eIF4B has only been identified to directly bind eIF3a in HeLa cells (63), components of eIF3 have been identified in stress granules, including eIF3b (64), and as 14 of the 115 eIF4B interacting proteins are core components of stress granules (Supplementary Figure S1B), we could postulate that these interactions could occur under specific conditions. Additionally, eIF3g is known to interact with SLIP1, which is bound to SLBP during histone mRNA translation (60), and eIF3 has been shown to bind histone mRNA stem–loops (65), so it is plausible that eIF4B could transiently interact with eIF3.

**Figure 6.**
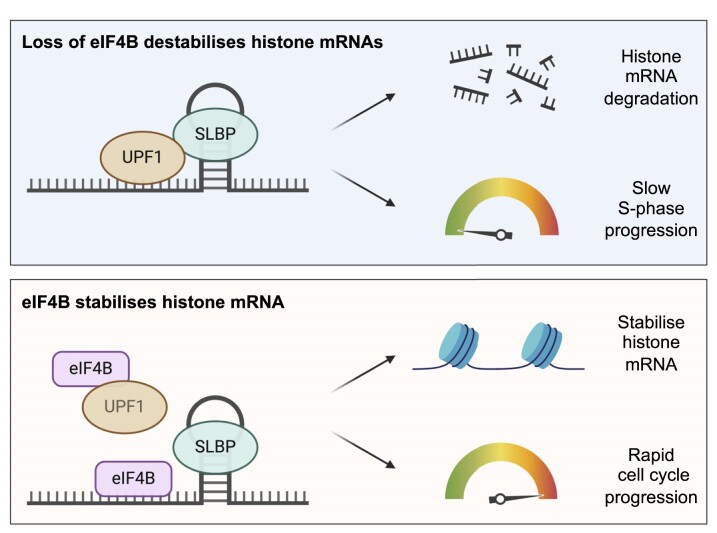
Proposed model of eIF4B regulation of histone mRNAs and cell cycle progression The loss of eIF4B leads to reduction in histone mRNA half-life and disrupted S-phase progression and cell proliferation. We propose that eIF4B stabilizes histone mRNAs by binding directly to histone mRNAs upstream of the stem–loop and to UPF1 protein, promoting rapid and accurate S-phase progression. Created with BioRender.com.

A previous screen in yeast suggested that eIF4B was not required for the translation of histone mRNAs or SLBP binding and function (66); however, our data indicate that eIF4B may function in this process and highlight a biological difference between mammalian and yeast cells in this regard. We show that cells depleted of eIF4B cannot progress readily through S phase and we hypothesize that eIF4B helps to stabilize histone mRNAs by binding directly to histone mRNAs upstream of the stem–loop and to UPF1 protein, providing a key link between translation and degradation of replication-dependent histone mRNAs (Figure 6).

However, we show that eIF4B has over 3000 target RNAs that are involved in essential cellular processes, including proliferative mRNAs such as CDK4, which mediates progression into S phase (67). Therefore, an alternative hypothesis could be that the loss of eIF4B generally disrupts cell cycle progression and proliferation, which could in turn dysregulate S-phase progression and histone mRNA levels. Although eIF4B knockdown did not alter the cell cycle profiles of asynchronous cells (Figure 5D), these were acute depletion experiments and it is known that protein synthesis rates are intricately linked to cell cycle state (68). Therefore, to fully ascertain the regulatory mechanism of eIF4B on histone mRNAs and S-phase progression, it would be beneficial to reintroduce eIF4B to rescue histone mRNA stability or to express mutant histone mRNAs that do not contain the eIF4B binding sites and function independently of eIF4B expression.

As the different genetic backgrounds of our tumour (DoHH-2) and non-tumour (GM01953) cell lines could contribute to the observed differences in eIF4B interactomes, direct comparisons should be made cautiously; however, these data do suggest that eIF4B is required for the efficient regulation of histone mRNAs to drive S-phase progression and provide further rationale for targeting high levels of eIF4B in DLBCL and other malignant diseases.

## Supplementary Material

gkae767_Supplemental_Files

## Data Availability

eIF4B co-immunoprecipitation mass spectrometry proteomics data have been deposited to the ProteomeXchange Consortium via the PRIDE (69) partner repository with the dataset identifier PXD042538. The sequencing data discussed in this publication have been deposited in NCBI’s Gene Expression Omnibus (70) and are accessible through GEO Series accession number GSE233998 (https://www.ncbi.nlm.nih.gov/geo/query/acc.cgi?acc=GSE233998).
