# Supplementary material for: Eukaryotic initiation factor 4B is a multi-functional RNA binding protein that regulates histone mRNAs
Source: Nucleic Acids Res. 2024 Sep 3;52(19):12039–54. doi: 10.1093/nar/gkae767 (PMC11514447; doi:10.1093/nar/gkae767)
Supplement: gkae767_Supplemental_Files [file gkae767_supplemental_files.zip › Supplementary Material.pdf]

Supplementary Figure 1

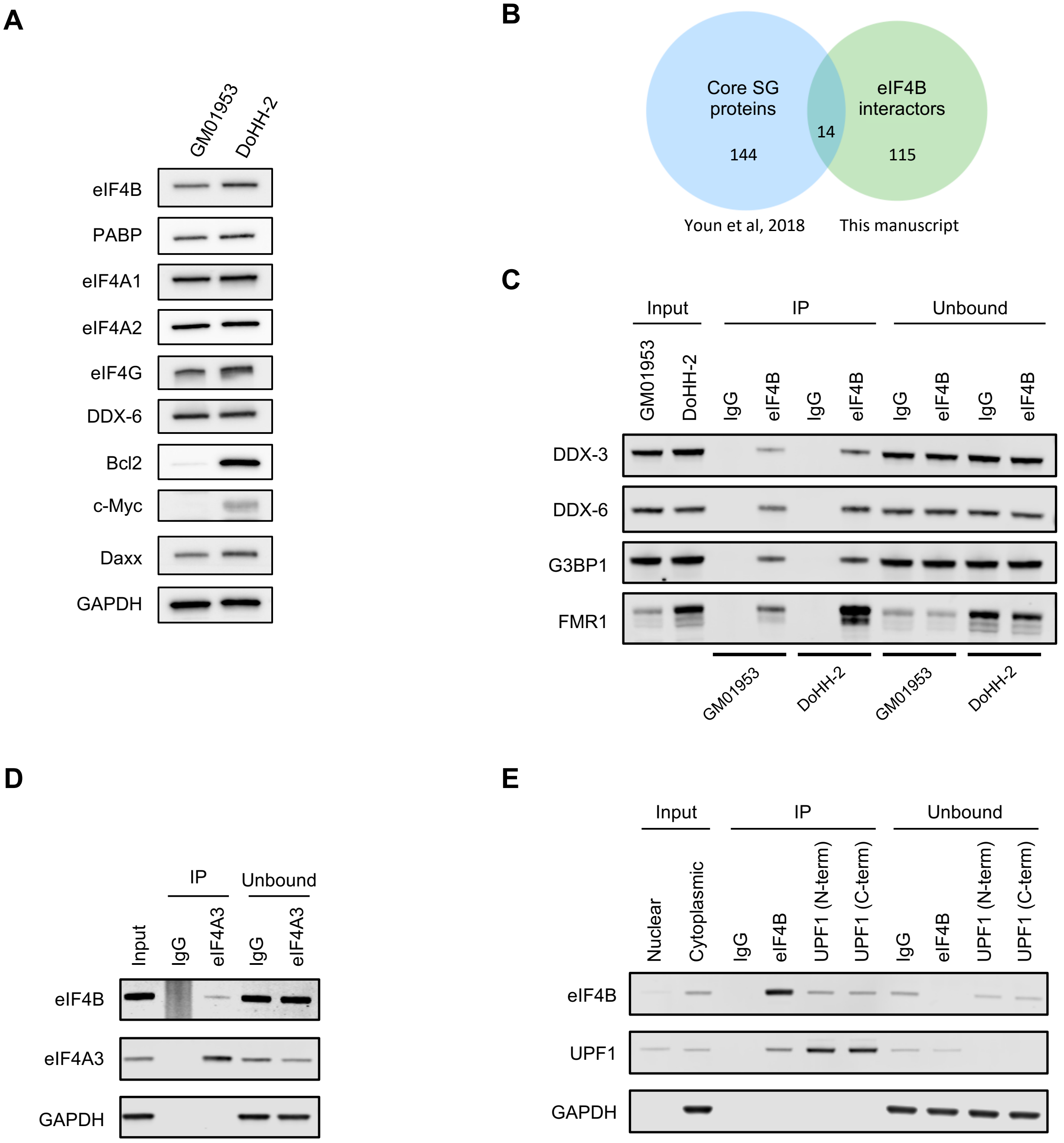

Supplementary figure 1. Identification of novel eIF4B interacting proteins involved in nonsense mediated decay.

A. Whole cell lysates were prepared from DoHH-2 (DLBCL) and GM01953 (non-tumour) cell lines and immunoblotted for the indicated antibodies. Blots shown are representative of three independent experiments.

B. Venn diagram of the overlap between our eIF4B interactome and proteins identified as core stress granule components (42).

C. As in figure 1D, cell lysates from DoHH-2 and GM01953 cells were immunoprecipitated (IP) for eIF4B or IgG. After RNA digestion and washing to remove RNA-dependent interactions, directly interacting proteins were analysed by immunoblotting with the indicated antibodies. Blots shown are representative of 3 independent experiments.

D. DoHH-2 whole cell lysates cells were immunoprecipitated (IP) for eIF4A3 or IgG. After RNA digestion, directly interacting proteins were analysed by immunoblotting with the indicated antibodies. Blots shown are representative of 3 independent experiments.

E. DoHH-2 cells were fractionated and the cytoplasmic enriched lysate was immunoprecipitated (IP) for eIF4B, IgG or UPF1 using antibodies that recognise either the N-terminal and C-terminal end of UPF1. After RNA digestion, directly interacting proteins were analysed by immunoblotting with the indicated antibodies. Blots shown are representative of 3 independent experiments.

Supplementary Figure 2

A

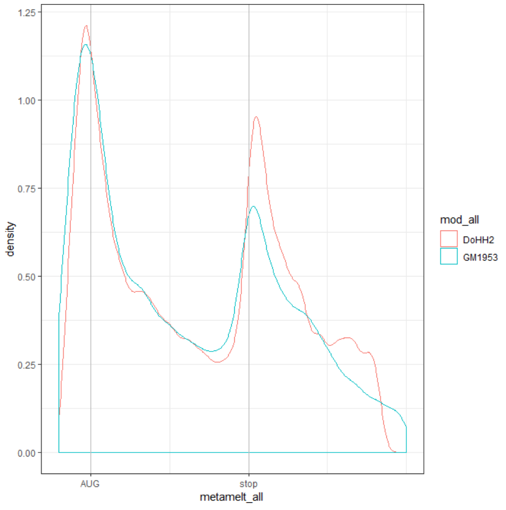

Supplementary Figure 2. Identification of eIF4B RNA binding sites by iCLIP in DLBCL.

A. Meta-analysis of eIF4B binding density across the length of all mRNAs identified with iCLIP in DoHH-2 and GM01953 cells.

Supplementary Figure 3

A

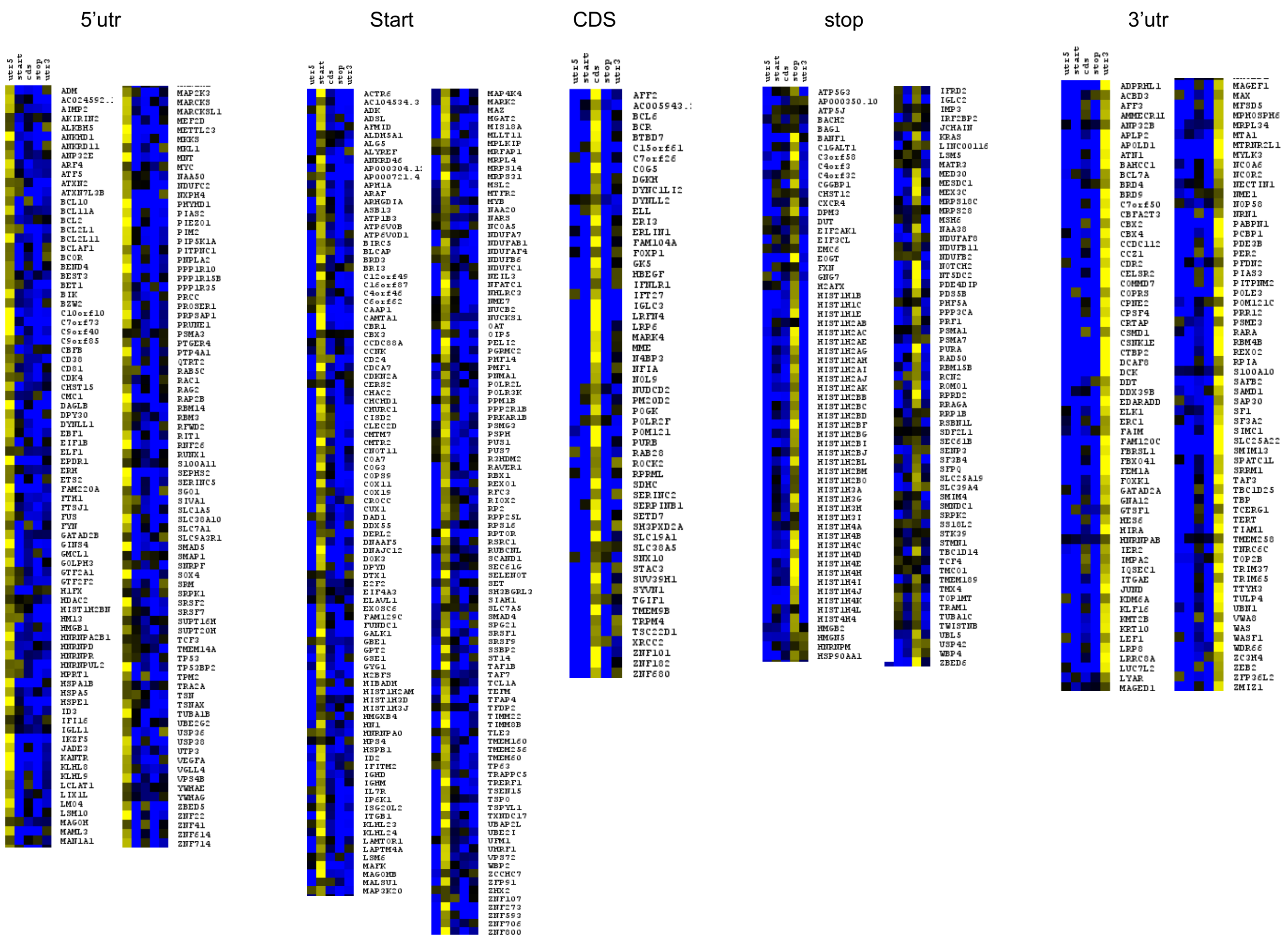

Supplementary Figure 3. eIF4B binds across the entire length of mRNA transcripts.

A. List of mRNAs classified within 5 distinct groups (from Figure 3A) based on the primary location of eIF4B binding (5'UTR, start codon, CDS, stop codon, 3' UTR).

Supplementary Figure 4

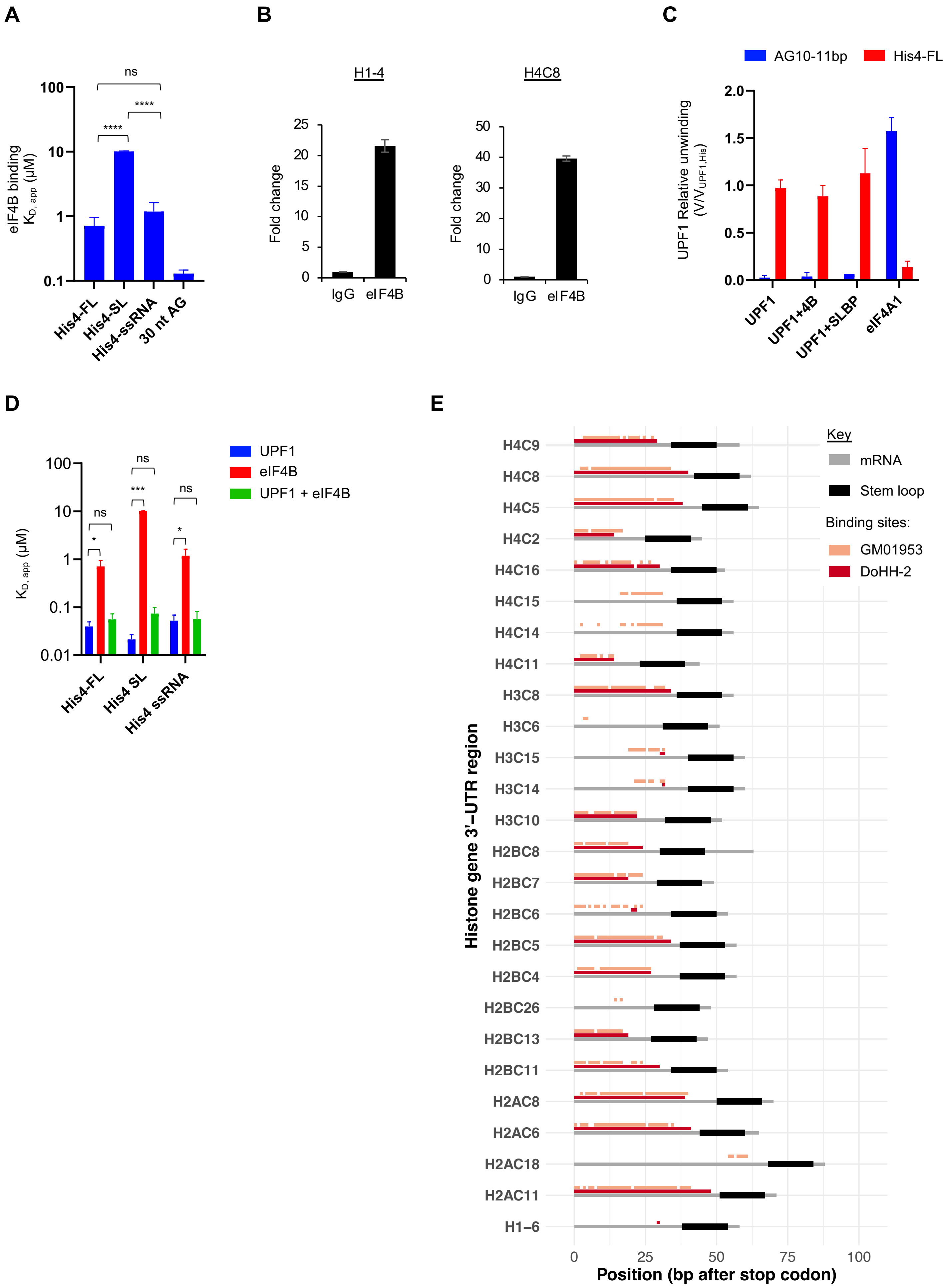

Supplementary Figure 4. eIF4B binds histone mRNAs.

A. Fluorescence-based RNA binding assay of eIF4B binding to tool RNAs based on histone 4 mRNA tool sequences (Supplementary table 4). Dissociation constants of eIF4B RNA binding to the indicated histone RNA sequence. Error bars represent standard deviation (n = 3 independent experiments); **** = P ≤ 0.001 by ANOVA. His4-FL: full length sequence, His4-SL: containing histone stem loop only, His4-ssRNA: full-length minus the stem sloop.

B. RIP qPCR of eIF4B in DoHH-2 cells. Binding of H1-4 and H4C8 mRNA to eIF4B was normalised to input RNA levels and shown relative to IgG control. Error bars represent mean ± standard deviation (n = 3 technical replicates).

C. Fluorescence-based RNA unwinding assay of a tool RNA sequence based on histone 4 mRNA stem loop. A linear regression model was used to determine velocity of unwinding for UPF1 alone, UPF1 in the presence of eIF4B, UPF1 in the presence of SLBP, and eIF4A1. As an additional control, unwinding velocity was determined for an eIF4A1 dependent sequence (AG10-11bp).

D. Fluorescence-based RNA binding assay of UPF1 and eIF4B binding to tool RNAs based on histone 4 mRNA tool sequences. Dissociation constants of binding to the indicated RNA sequence by UPF1 alone (blue), eIF4B alone (red) and UPF1 in the presence of eIF4B (green). Error bars represent standard deviation (n = 3 independent experiments); * = $P \leq 0.05$, *** = $P \leq 0.001$ by ANOVA. His4-FL: full length sequence, His4-SL: containing histone stem loop only, His4-ssRNA: full-length minus the stem sloop.

E. Comparison of eIF4B iCLIP binding sites from DoHH-2 (red) and GM01953 (peach) on histone mRNAs that were only bound by eIF4B in iCLIP analysis from DoHH-2 (red) and GM01953 (peach) cells. All mRNAs (grey) were aligned at the stop codon and the stem loop location is indicated in black.

Supplementary Figure 5

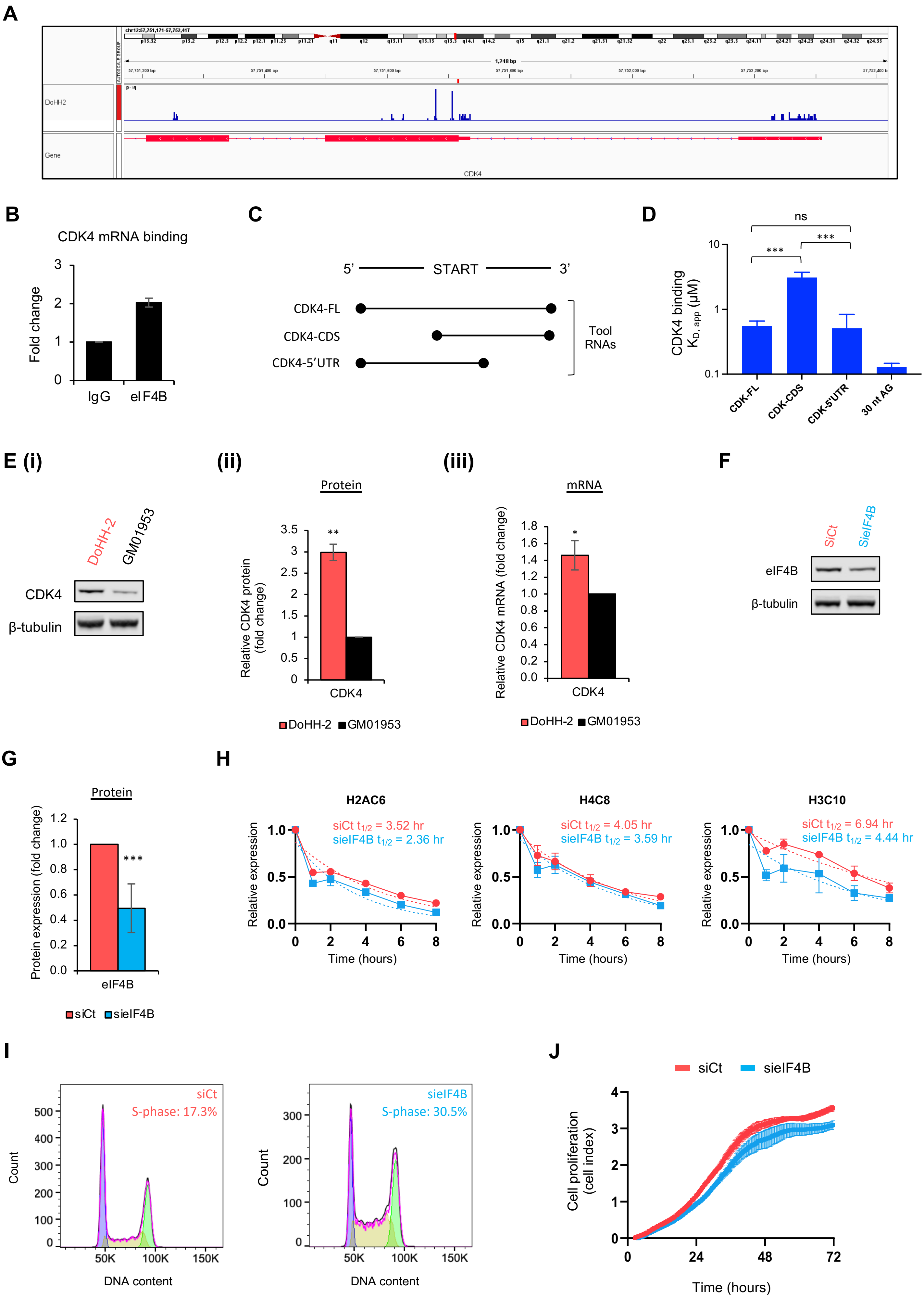

Supplementary Figure 5. eIF4B stabilises histone mRNAs and accurate S-phase progression.

A. Visualisation of eIF4B binding to the 5'UTR of CDK4 mRNA using the integrative genomics viewer (IGV) [57]. eIF4B iCLIP binding peaks from DoHH-2 cells are shown in blue.

B. RIP qPCR of eIF4B in DoHH-2 cells. Binding of CDK4 mRNA to eIF4B was normalised to input RNA levels and shown relative to IgG control. Error bars represent mean ± standard deviation (n = 3 technical replicates).

C. Schematic representation of tool RNAs (Supplementary table 4) based on CDK4 mRNA. CDK4-FL: full length sequence, CDK4-CDS: encompassing start codon and CDS region, CDK4-5'UTR: encompassing 5'UTR and start codon.

D. Fluorescence-based RNA binding assay showing dissociation constants of eIF4B RNA binding to tool RNAs based on CDK4 mRNA tool sequences from (C) and a 30 nt AG positive control for eIF4B binding. Error bars represent mean ± standard deviation (n = 3 independent experiments); *** = $P \leq 0.001$ by ANOVA.

E. (i) Whole cell lysates were prepared from DoHH-2 and GM01953 cell lines and immunoblotted for the indicated antibodies. Blots shown are representative of three independent experiments. (ii) Quantification of CDK4 protein expression from (i) normalised to β-tubulin. Mean levels from DoHH-2 are displayed as a fold change relative to GM01953. Error bars represent mean ± standard deviation (n = 3 independent experiments); ** = $P \leq 0.01$ by unpaired Student's t test. (iii) Total RNA was isolated from DoHH-2 and GM01953 cells in parallel to (i). cDNA was synthesised and qPCR was used to quantify CDK4 mRNA levels. CDK4 mRNA expression levels in DoHH-2 cell are displayed as a fold change relative to GM01953 levels. Error bars represent mean ± standard deviation (n = 3 independent experiments); * = $P \leq 0.05$ by unpaired Student's t test.

F. DoHH-2 cells were transfected with a control non-targeting siRNA or an siRNA specific for eIF4B (100 nM) for 72 hours. Cells were lysed and knockdown efficiency was determined by immunoblotting with the indicated antibodies. Blots are representative of three independent experiments.

G. Quantification of eIF4B protein levels from (F) normalised to β-tubulin levels. Mean levels from sieIF4B are displayed as a fold change relative to siCt. Error bars represent mean ± standard deviation (n = 3 independent experiments); *** = $P \leq 0.001$ by unpaired Student's t test.

H. mRNA stabilisation assay in parallel to Figure 5B. Estimation of mRNA half-life (t1/2) was determined using a one-phase decay model (dotted line). Mean values for each time point are shown (solid line) and error bars represent standard deviation (n = 3 independent experiments).

I. A representative example of cell cycle quantification using Watson (pragmatic) modelling from the representative siCt (left) and sieIF4B (right) cell cycle profiles from Figure 5E.

J. HeLa cell proliferation from the xCELLigence RTCA DP instrument following siRNA knockdown of eIF4B (30 nM) for 48 hr. Proliferation was monitored up to 72 hours. Cell index is an arbitrary value derived from the xCELLigence RTCA DP instrument measuring electrical impedance of cells and is an indicator of cell number. Data shows a representative experiment and error bars represent mean ± standard deviation (n = 4).
