# Supplementary material for: Eukaryotic initiation factor 4B is a multi-functional RNA binding protein that regulates histone mRNAs
Source: Nucleic Acids Res. 2024 Sep 3;52(19):12039–54. doi: 10.1093/nar/gkae767 (PMC11514447; doi:10.1093/nar/gkae767)
Supplement: gkae767_Supplemental_Files [file gkae767_supplemental_files.zip › Supplementary table legends.docx]

**Supplementary table legends**

**Supplementary table 1.** iCLIP primers used for RNA reveres transcription.

**Supplementary table 2.** eIF4B co-immunoprecipitation mass spectrometry analysis in DoHH-2 and GM01953 cells.

**Supplementary table 3.** iCLIP analysis and eIF4B binding clusters.

**Supplementary table 4.** In vitro binding tool RNA sequences

**Supplementary table 5.** Comparison of eIF4B and UPF1 binding to histone mRNAs.
